# Rives Technique for the Primary Larger Inguinal Hernia Repair: A Prospective Study of 1000 Repairs

**DOI:** 10.1007/s00268-017-4038-z

**Published:** 2017-05-08

**Authors:** Enrique J. Grau-Talens, Carlos D. Ibáñez, Jacob Motos-Micó, Francisco García-Olives, Martina Arribas-Jurado, Carlos Jordán-Chaves, José M. Aparicio-Gallego, José F. Salgado

**Affiliations:** 1 Hospital Siberia-Serena, Carretera Talarrubias-Agudo, SN, 06640 Talarrubias, Badajoz Spain; 2 Hospital Siberia-Serena, Calle Zafra, 3, 3D, 06400 Don Benito, Badajoz Spain; 3 Hospital Siberia-Serena, Calle Canaleja, 4, 2º A, 06400 Don Benito, Badajoz Spain; 4 Verge Del Toro Hospital, Menorca. Fort de L’eau 96, 07701 Mahón, Balearic Islands Spain; 5 Calle La Cola, 10, 14300 Villaviciosa de Córdoba, Córdoba Spain; 6 Hospital Siberia-Serena, Calle Santiago, 32, 06900 Llerena, Badajoz Spain; 7 Calle 1º de mayo, 71, 4º, 06400 Don Benito, Badajoz Spain; 8 Calle Castillo 15, 06006 Zalamea de la Serena, Badajoz Spain

## Abstract

**Objective:**

We report a prospective study of repairs using the Rives technique of the more difficult primary inguinal hernias, focusing on the immediate post-operative period, clinical recurrence, testicular atrophy, and chronic pain. A mesh placed in the preperitoneal space can reduce recurrences and chronic pain.

**Methods:**

For the larger primary inguinal hernias (Types 3, 4, 6, and some 7), we favour preperitoneal placement of a mesh, covering the myopectineal orifice by means of a transinguinal (Rives technique) approach. The Rives technique was performed on 943 patients (1000 repairs), preferably under local anaesthesia plus sedation in ambulatory surgery.

**Results:**

The mean operative time was 31.8 min. Pain assessment after 24 h with an Andersen scale and a categorical scale gave two patients with intense pain on the Andersen scale, and four patients who thought their state was bad. Surgical wound complications were below 1%, and urinary retention was 1.2% mostly associated with spinal anaesthesia and, in one case, bladder perforation. There was spermatic cord and testicular oedema with some degree of orchitis in 17 patients. The clinical follow-up of 849 repairs (86.4%), mean (range) 30.0 (12–192) months, gave five recurrences (0.6%), three cases (0.4%) of testicular atrophy, and 37 (4.3%) of post-operative chronic pain (8 patients with visual analogue scale of 3–10).

**Conclusions:**

The Rives technique requires a sound knowledge of inguinal preperitoneal space anatomy, but it is an excellent technique for the larger and difficult primary inguinal hernias, giving a low rate of recurrences and chronic pain.

## Introduction

The value of a surgical intervention lies in the short and long term results (quality) provided at a reasonable cost [1]. The primary repair of an inguinal hernia aims to cure the symptoms and prevent immediate post-operative complications, chronic pain, and recurrences. Mesh repair has reduced the recurrence of inguinal hernias, but there is still a rate of 4% of recurrence and 12% of post-operative chronic pain, moderate-to-severe, with the Lichtenstein procedure [2].

The aim of our work is the prospective study of the Rives technique for repair of the larger primary inguinal hernias (transinguinal insertion of a mesh in the preperitoneal space), focusing on the immediate post-operative period, clinical recurrence, and chronic pain. The mesh placed in the preperitoneal space, covering the myopectineal orifice of Fruchaud and separated from the neurological plane, is the ideal location hydrostatically and anatomically to prevent the recurrence of the three potential hernia orifices and chronic pain.

## Patients and methods

From January 1992 to April 2014, we performed 1000 primary inguinal hernia repairs in 943 patients using the Rives technique: 198 patients at the Verge del Toro Hospital, Mahon, Menorca (Balearic Islands), Spain (1992–2001); and 745 patients from October 2007 until April 2014 in ambulatory/short-stay surgery at the Siberia-Serena Hospital, Talarrubias (Badajoz), Spain. In 1994, all the data of the operated patients, which had been collected in paper form, were input into an Access-form database. All data collected thereafter were input into the said database on a daily basis and analysed with Excel.

Between 2001 and 2006, the first author did not perform abdominal wall surgery because he was assigned to the coloproctology section of the Infanta Cristina University Hospital, Badajoz, Spain.

### Inclusion criteria and perioperative measures and follow-up

We use Gilbert’s classification with additions by Rutkow and Robbins [3]. The Rives technique was used in Types 2 (when the deep inguinal ring was about 4 cm in diameter), 3, 4, 6, and some of the 7.

In ambulatory surgery, all patients are contacted via telephone within 24 h of surgery and pain is evaluated at rest and mobilization using the Andersen scale [4]. This is concluded with a subjective assessment by the patient on a categorical scale of bad, fair, good, or excellent overall condition. After a week they are called again.

The first 467 patients were clinically reviewed in the office within 7–10 days of discharge (for the rest of the patients, we opted for telephone inquiry to spare them from coming to the hospital, where the wound was observed, and pain was evaluated at rest and with movement (standing up, sitting down, and walking), using a VAS (visual analogue scale) in 154 consecutive patients, as a representative sample size for post-operative pain.

They are reviewed clinically by the service’s surgeons again after a year or earlier if the patient experiences pain, and called again 3 or more years later. If pain is found, this is evaluated as being with movement, spontaneous, episodic, or constant in nature, and measured with a VAS. The inguinal region is examined. A mass, reducible or otherwise, is regarded as a recurrence. Testicular examination is made for atrophy or other pathology, and inquiry for late infection.

Preoperative preparation includes the prophylaxis of nausea and vomiting with dexamethasone 8 mg IV. Prophylaxis of the surgical site infection (cefazoline 2 g IV) is not performed routinely. It was done in obese, diabetics, and bilateral hernia repair. Glove change and wound protection with gauze before inserting the mesh were the rule. Lastly, rinsing wound preceded the skin sutures.

### The technique now

We perform the technique preferably under local anaesthesia and sedation, with mepivacaine 1%, 400–500 mg (6 mg/kg) according to the technique of Ponka and Flanagan [5]. An anaesthesiologist provides sedation. The sedation begins with midazolam (0.03 mg/kg IV) followed by propofol (25–50 mc/kg/min). Upon finishing the intervention, we infiltrate the surgical field with 20–30 ml of 0.25% bupivacaine. In very large inguinoscrotal or non-reducible hernias, spinal or general anaesthesia is used.

**Fig. 1:**
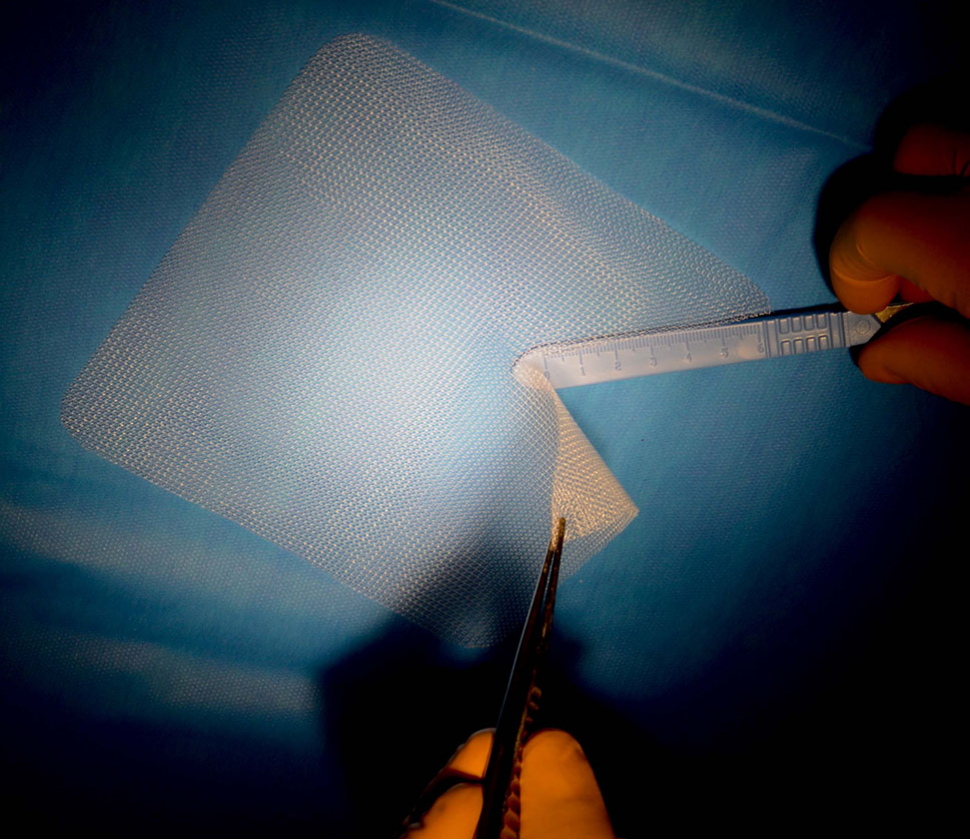
Slit for the spermatic cord

The surgical technique comprises the insertion of a mesh in the preperitoneal space through the groin as described by Rives [6–10]. We use a 15 × 15 cm polypropylene mesh (standard weight 80 g/m^2^, and macro-porous 0.5 × 0.7 mm) in which we make a slit for the passage of the spermatic cord (Fig. 1). We have made some modifications over the years, but we remain faithful to the principles of the technique.

**Fig. 2:**
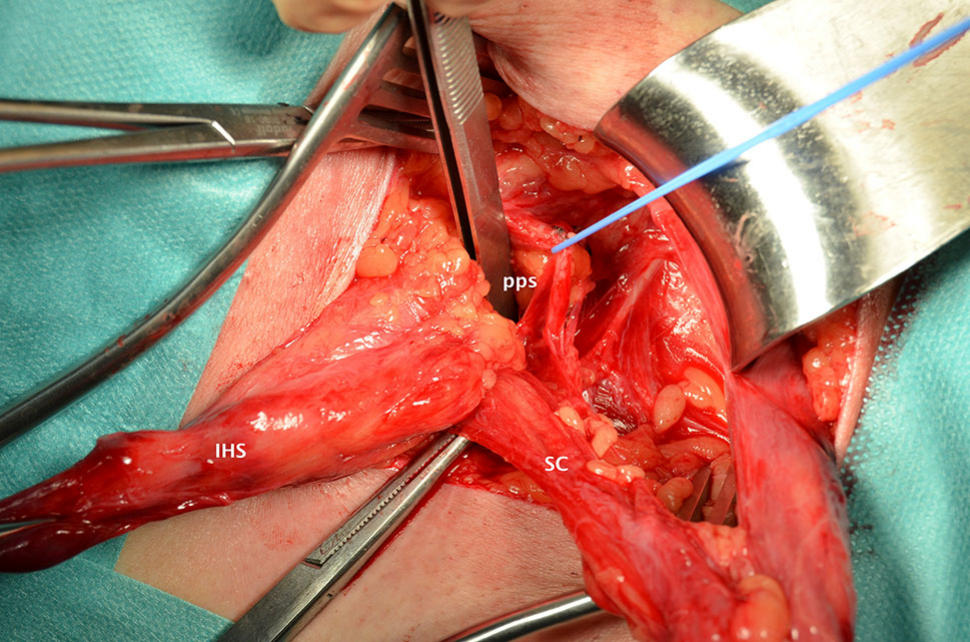
DIR (deep inguinal ring) is dissected. A vessel loop is pulling the inferior epigastric vessels. Forceps are in the preperitoneal space (PPS). *IHS* indirect hernial sac. *SC* spermatic cord

An incision of 6–8 cm in length is made in the medial 2/3 of the line from the anterior superior iliac spine to the pubic tubercle. The ilioinguinal, iliohypogastric, and genital branch of the genitofemoral nerve are preserved. The cremaster is not removed, but opened longitudinally and split to separate the fibres along with the external spermatic vessels (blue line) [10, 11] and the genital branch of the genitofemoral nerve. The spermatic cord, without the cremaster, is lifted and held with a rubber band. The internal spermatic fascia is opened up to the preperitoneal fat (space of Bogros), and the indirect sac (and lipomata) is dissected up to the peritoneal level (true neck) in the deep inguinal ring (DIR) [12] and reduced to the iliac fossa (Fig. 2).

**Fig. 3:**
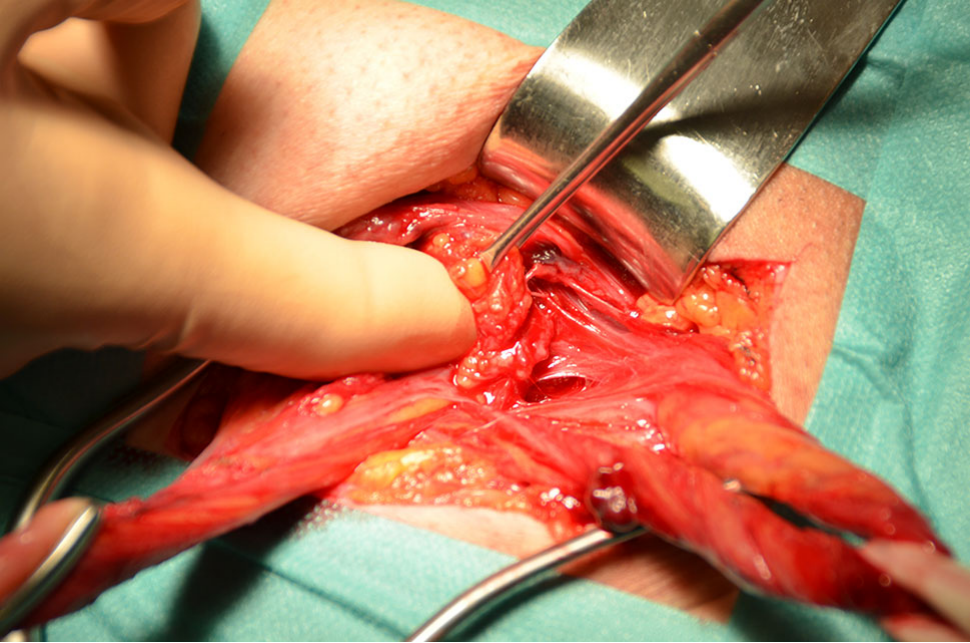
Preperitoneal space for mesh accommodation is created with the finger

**Fig. 4:**
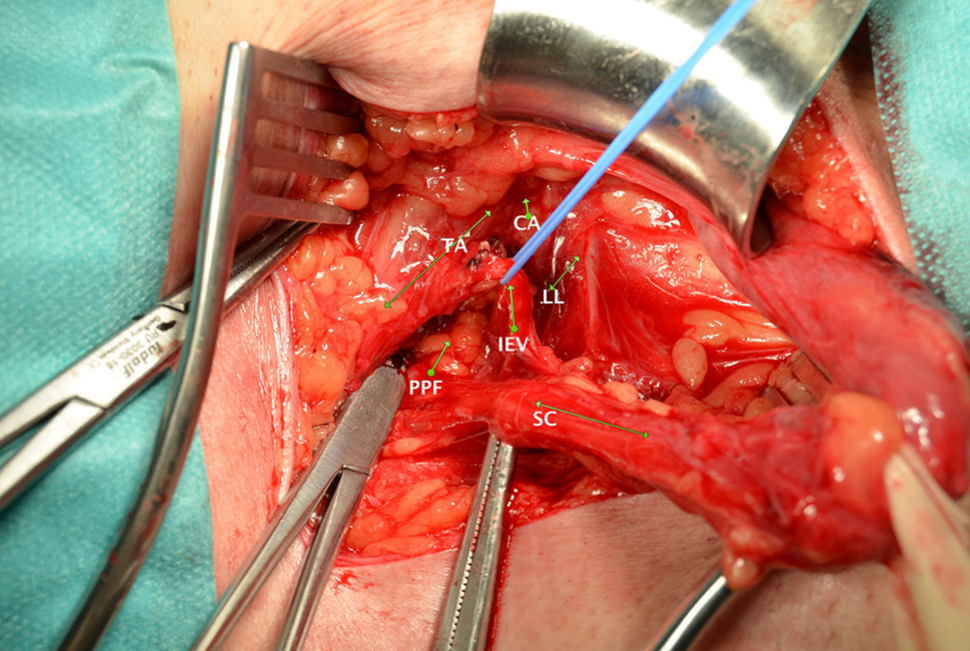
Anatomic vision of the DIR in an indirect large hernia. The indirect hernia sac is already reduced. *TA* transversus abdominis arch. *CA* conjoined area. *LL* lacunar ligament. *IEV* inferior epigastric vessels. *SC* spermatic cord. *PPF* Preperitoneal fat

**Fig. 5:**
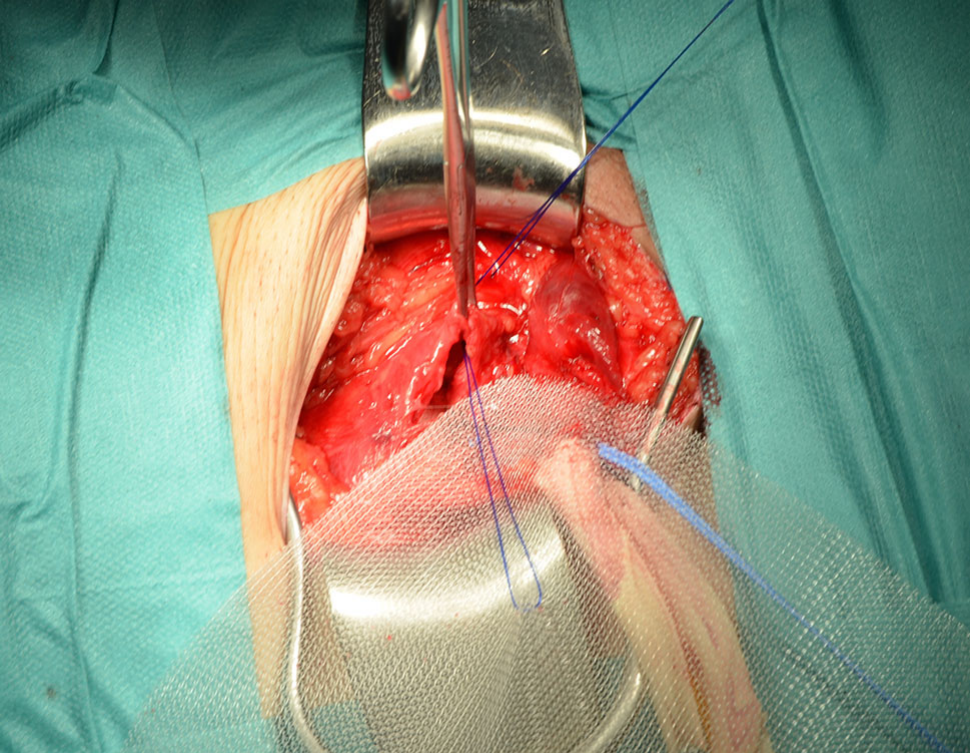
Mesh ready to be passed under epigastric vessels to lie under conjoined area. A stitch running through the conjoined area near the pubic tubercle, coming out through the DIR under the inferior epigastric vessels, takes the mesh and returns the same way it came to the conjoined area

*Indirect hernia* At the medial edge of the DIR are the inferior epigastric vessels displaced medially, and posterior to them and to the transversalis fascia (Retzius space) we create a space, by sharp and blunt dissection, to accommodate the mesh (Figs. 3, 4). Cooper’s ligament can be seen, and the femoral orifice is explored for a femoral hernial sac. The mesh is introduced through the DIR with a stitch passed through the conjoined tendon, emerging through the DIR posterior to the inferior epigastric vessels, and taking the mesh with it as it returns, coming out again through the conjoined tendon (Fig. 5).

**Fig. 6:**
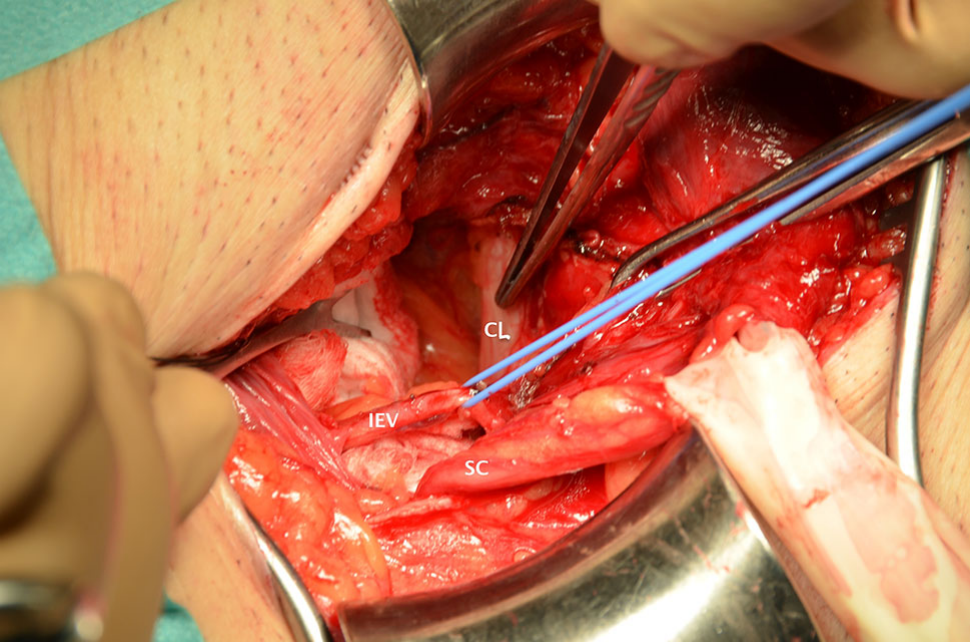
Transversalis fascia over a direct sac has been opened and the hernia reduced. A wound swab is introduced. Forceps are pointing at the Cooper ligament (CL). Inferior epigastric vessels (IEV) are pulled by a vessel loop. The spermatic cord (SC) is held with a rubber band

*In direct hernias* (the DIR is explored for an indirect sac) the attenuated transversus abdominis aponeurosis and transversalis fascia over the direct sac are opened transversally to enter the preperitoneal space. A femoral sac, if any, is reduced. The inferior epigastric vessels are released and preserved (Fig. 6), and the mesh, inserted through Hesselbach’s triangle, slides (preferably) under them or pushes them down and overlies them (less desirable case) [13].

**Fig. 7:**
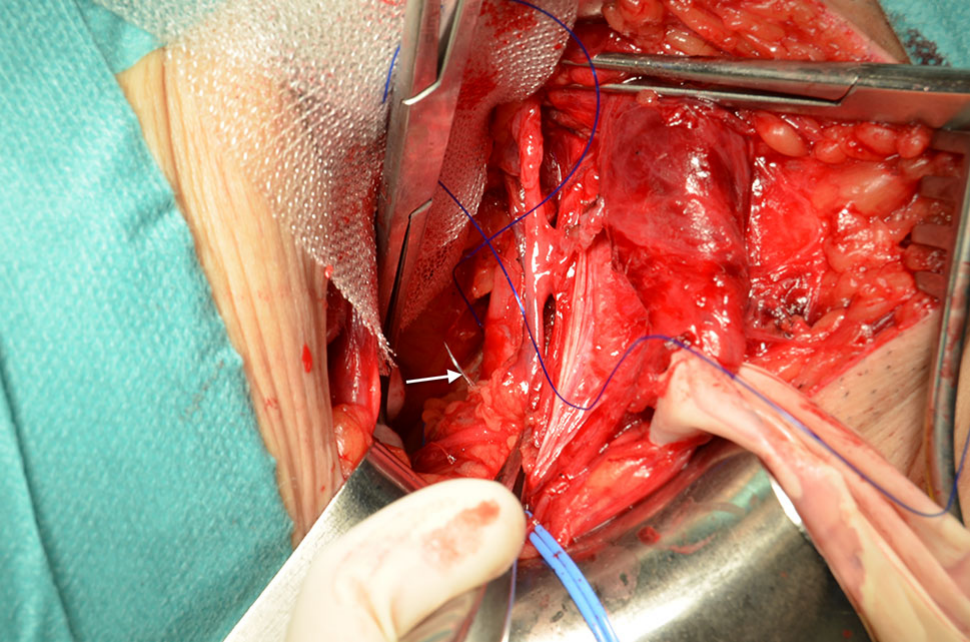
A stitch (*white arrow*) is passed through Cooper ligament

*Mesh fixation* The mesh is fixed with 5–7 stitches of polypropylene monofilament with an appropriate needle (premilene^®^ No. 0, HR80, B Braun). The first stitch in the conjoined tendon (as described above) acts as an anchor. The second stitch takes the Cooper’s ligament (close to the femoral ring) and the mesh, at approximately 4 cm from the edge (Fig. 7), slid over it and covering the femoral and obturator rings. Particular attention has to be paid to the anastomotic pubic branch, and sometimes to the anterior pubic branch artery, obturator accessory, or aberrant artery and corresponding veins. In indirect hernias, due to the limited space of the DIR, it is easier to put the stitch into the Cooper´s ligament before mesh introduction and fixation to the posterior face of the conjoined tendon. If access to the Cooper ligament is difficult, the mesh is attached to the iliopubic tract (less desirable), with care taken of the inferior epigastric vessels.

**Fig. 8:**
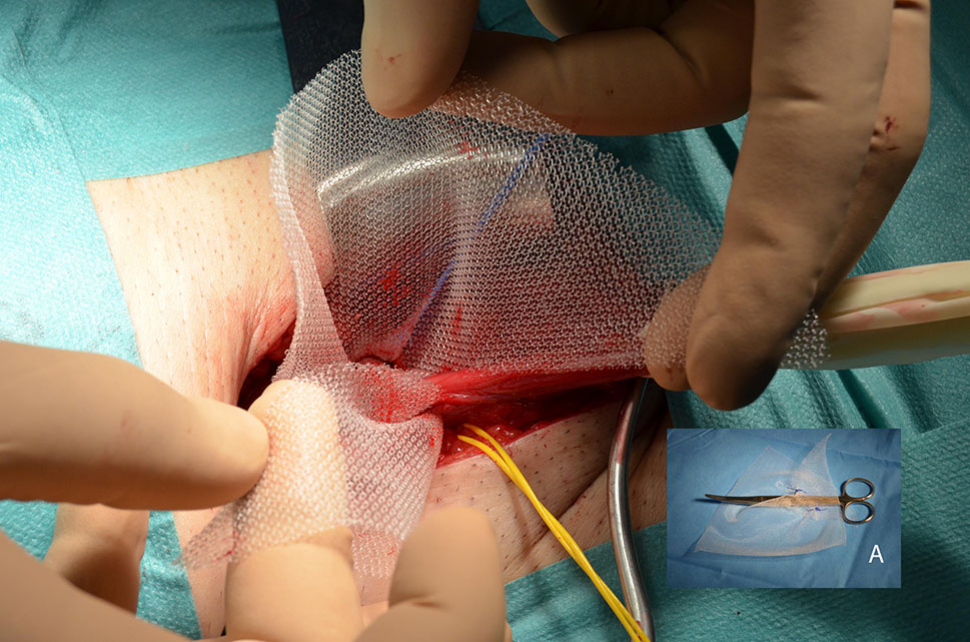
Spermatic cord is positioned in the slit and a tunnel-like flap is made around it, as seen in the *lower* image A

**Fig. 9:**
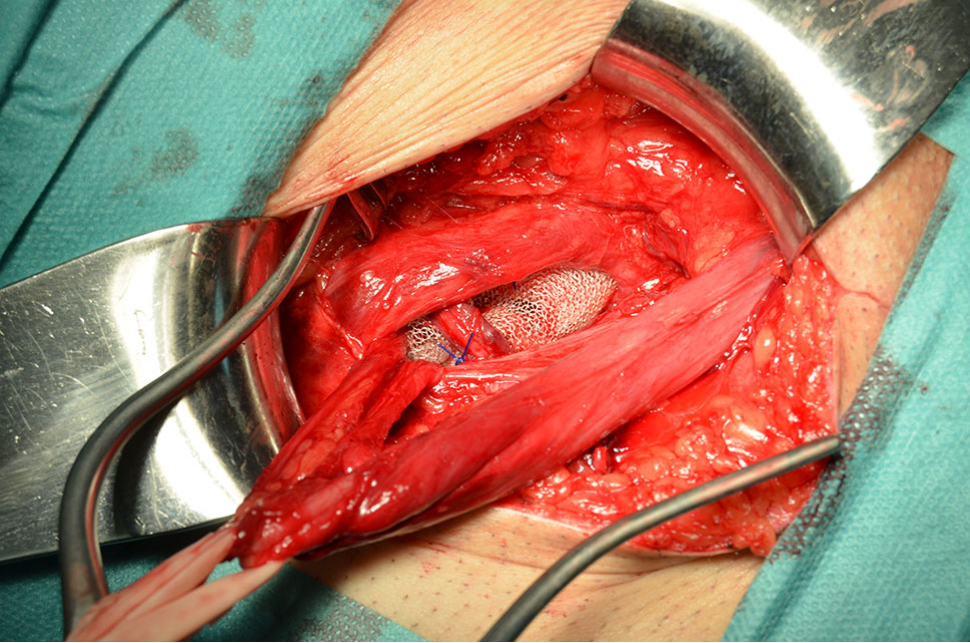
Mesh is in the preperitoneal space covering the whole myopectineal orifice in a type 6 (pantaloon) hernia

The spermatic cord is positioned in the slit of the mesh, and wrapped with it in the manner of a tunnel (Fig. 8). The mesh is slipped with Crile forceps behind the transversus abdominis muscle in the preperitoneal space. The third stitch fixes the mesh to the vascular fascia/iliopubic tract, taking care with the genital branch of the genitofemoral nerve, the fourth stitch is placed into the transversus abdominis arch, and the fifth into the most lateral zone of the transverse arch. Additional sutures are inserted if needed. The mesh covers and exceeds entirely, by at least 4 cm, the myopectineal orifice (Fig. 9).

## Results

The demographic and operative findings are presented in Tables 1 and 2. Pain assessment after 24 h in 761 consecutive unilateral repairs in ambulatory surgery (Table 3) revealed two patients with intense pain, while four patients referred to a bad state due to coughing (1), wound pain and testicular oedema (2), and urinary retention (1).

**Table 1:** Demographics of the primary inguinal hernia patients with Rives technique repair

Number of repairs	1000
Number of patients	943
Women/men	56/887
Age, mean (range) years	60.2 (18–93)
Body mass index, mean (SD)	28.2 (3.7)
BPH^a^, *n* (%)	164 (18.4%)
COPD^b^, *n* (%)	68 (7.2%)
Right/left	521/479
Repairs in day-surgery	785
Bilateral repair–simultaneous (*n* patients)	24
Bilateral repair–in two sessions (*n* patients)	19

^a^Benign prostatic hypertrophy

^b^Chronic obstructive pulmonary disease

**Table 2:** Operative findings in 1000 Rives repairs

Type 2, *n* (%)	36 (3.6)
Type 3, *n* (%)	564 (56.4)
Type 4, *n* (%)	321 (32.1)
Type 6, *n* (%)	45 (4.5)
Type 7, *n* (%)	8 (0.8)
Type 7 with another (direct or indirect), *n* (%)	26 (2.6)
Femoral hole **>**2 cm, *n* (%)	29 (2.9)
Duration of the intervention, mean (SD) min^a^	31.8 (7.5)
*Type of anaesthesia*	
General, *n* (%)	8 (0.8)
Spinal, *n* (%)	130 (13)
Local + sedation, *n* (%)	862 (86.2)

^a^From incision to the last stitch in the skin

**Table 3:** Andersen scale and overall categorical status assessment in 761 unilateral repairs in day-surgery at 24 h

	*N* (%)
0. No pain	291 (38)
1. Absence of pain at rest, mild with mobilization or cough	302 (40)
2. Mild at rest or moderate pain with mobilization or cough	139 (18)
3. Moderate at rest or intense pain with mobilization or cough	23 (3)
4. Intense pain at rest and extreme with mobilization or cough^a^	1 (0.1)
5. Very intense pain at rest^a^	1 (0.1)
Excellent, *n* (%)	239 (32)
Good, *n* (%)	456 (61)
Fair, *n* (%)	57 (7)
Bad, *n* (%)	4 (0.5)
No reply^b^	4

^a^Followed and asymptomatic

^b^Revised, without any incidents. One patient admitted for bleeding

The results of the VAS evaluation in the office 1 week after the intervention in 154 consecutive patients were 0.5 (1.5) at rest, and 2.7 (2.1) in movement [mean (SD)].

Surgical wound complications (Table 4) were below 1%. One patient suffered post-operative hypotension with a drop in haemoglobin levels and was re-operated with the finding of a large bleeding hernial sac without associated vascular lesion. One bladder perforation occurred due to an unusual adherence of the bladder to a fibrotic DIR. Spermatic cord and testicular oedema with some degree of orchitis was detected in 17 patients. Three patients presented testicular atrophy.

**Table 4:** Post-operative complications in 1000 Rives technique repairs

	*N* (%)
Haematoma^a^	7 (0.7)
Seroma	9 (0.9)
Infection^b^	9 (0.9)
Sinus	1 (0.1)
Bladder injury	1 (0.1)
Urinary retention	12 (1.2)
Testicular pain and swelling	17 (1.7)
Re-operation^c^	1 (0.1)
Death	0

^a^Four admitted for observation, and three evacuated and sutured

^b^One patient with Fournier gangrene

^c^For a larger bleeding hernial sac

Of the 1000 repairs, 16 patients died in the first year, so that 984 repairs were called for clinical review, and 849 repairs (86.4%) were actually clinically reviewed. The overall results of the follow-up are given in Table 5. No late infections of the repair were detected.

**Table 5:** Overall clinical follow-up of 984 Rives technique repairs

Followed up, *n* (%)	849 (86.4)
Mean (range), month	30.0 (12–192)
Median, month	23
Recurrence, *n* (%)	5 (0.6)
Testicular atrophy, *n* (%)	3 (0.4)
Hydrocele, *n* (%)	6 (0.7)
Pseudocyst of spermatic cord, *n* (%)	5 (0.6)

The chronic pain measurements registered after 1 year are presented in Table 6. Six patients were treated by infiltration, of whom four had major improvement of the pain. The remaining two needed surgical revision and neurectomy for pain resolution. Currently, there is no patient with severe pain in our series.

**Table 6:** Chronic pain at 1 year

Total, *n* (%)		37 (4.3)
Pain in movements, *n* (%)		17 (2.0)
	VAS^a^ < 3, *n*	11
	VAS = 3–6, *n*	3
	VAS = 6–10, *n*	3
Pain episodic spontaneous, *n* (%)		22 (2.5)
	VAS < 3, *n*	21
	VAS = 3–6, *n*	2
Pain constant, *n* (%)		2 (0.3)
	VAS < 3, *n*	2

^a^ *VAS* visual analogue scale

## Discussion

All surgeons who operate inguinal hernias must have deep knowledge of the inguinal region’s anatomy, and this is especially true for the Rives repair technique [12–17].

The preperitoneal space is the most suitable site for the insertion of a mesh [2] because the hydrostatic pressure of the abdominal cavity itself will fix the mesh against the abdominal wall, provided there is sufficient mesh extension (4 cm) around the hernial ring. There is less intra-abdominal pressure on the mesh if it is located in the preperitoneal space due to the smaller radius (Laplace’s law).

The internal spermatic fascia is a continuation of the fascia transversalis [14], and in indirect hernia the dissection of the hernial sac must be prolonged until the preperitoneal fat appears. Medially to the true deep inguinal ring, the inferior epigastric vessels can be seen more or less clearly enveloped in a condensation of transversalis fascia. Sharp dissection, opening the scissor’s tip posterior to the epigastric vessels, allows for the exploration of the wall posterior to the transversalis fascia. This sharp dissection is likely to be necessary because the Retzius space is laterally closed along the length of the inferior epigastric vessels [16, 18]. The preperitoneal fat must emerge clearly behind the epigastric vessels to allow the exploration, without risk or difficulty, of the Bogros and Retzius spaces.

The inferior epigastric vein and artery are spared. In a few cases of difficult hernias in elderly patients, they were ligated to improve repair. The presence of communicating arteries, aberrant or accessory obturator, which may be as frequent as 30–69% [19], must be demonstrated by blunt dissection, and, generally speaking, it is easy to avoid puncturing the vessels. Occasionally it is risky to insert a stitch into the Cooper’s ligament, and then we settle for the iliopubic tract and the femoral sheath.

The lymphoadipose tissue can simulate the presence of a hernial sac in the femoral canal, and when in doubt we explore the subinguinal region to rule out the presence of a sac in the oval fossa.

Independently of the existence of a clear femoral hernia, the presence of a wide femoral ring arouses suspicions of a future femoral hernia [20]. Nevertheless, given that an epigastric hernia can develop through a ring only a few millimetres wide, the femoral ring’s diameter loses importance, while the need to protect it from future hernias with a prosthesis anchored in Cooper’s ligament gains in strength.

In Lichtenstein’s technique, the femoral ring is strengthened when judged large, while in the Rives technique this is done systematically. Between 7.9 and 40% [21–23] of the recurrences in an inguinal hernia occur through the femoral ring. Whether they are really recurrences or rather undiscovered hernias is unknown. In any case, Mikkelsen’s study’s [21] main finding was a 15-fold greater incidence of femoral hernia repair in patients who had previously had an inguinal hernia repair compared with the spontaneous operation rate for a femoral hernia. The rate of recurrences after Lichtenstein’s operation is taken by the European Hernia Society [2] to be 4%, although the reality is probably different [24] with a rate twice or thrice that. Even if hernia recurrence has indeed dropped, we still cannot be sure that it is not the main concern in the repair of a primary inguinal hernia. Our recurrence rate of 0.6% is within the range (0–1.2%) of those of Read [9], Pélissier et al. [25], and Koning et al. [26, 27]. However, we believe that recurrence should be zero or anecdotal for a technique that covers all potential hernial orifices and turns the slit into a flap for the passage of the spermatic cord avoiding indirect recurrences. The Nyhus posterior preperitoneal prosthetic placement has a similar conception to the transinguinal way and has provided with excellent results when the prosthesis was extended to buttress the abdominal wound [28, 29].

Post-operative chronic pain is defined by the International Guidelines as a pain that was absent prior to the intervention or is different from that prior to the intervention, and lasts for more than 3 months [2]. Franneby et al. [30] found 30% of inguinal herniorrhaphy patients who reported pain or discomfort and nearly 6% high-intensity pain with inability to perform daily activities. There is a generalized consensus that identification and protection of the three inguinal nerves lowers the risk of developing severe chronic post-operative pain [11, 30, 31]. From the results of a double-blind, randomized controlled trial, Mui et al. [32] advocated that prophylactic ilioinguinal neurectomy should be incorporated into the essential steps of Lichtenstein hernia repair.

The incidence of chronic pain of any kind in our patients after 1 year was 4.3% and moderate/severe pain 1.0%. With these results, we can conclude that the polypropylene mesh in preperitoneal position and preservation of the three nerves gives a low chronic post-operative pain rate. However, we needed two neurectomies and four infiltrations for there to be no patient with any pain greater than some mild discomfort.

There are simpler options for inguinal hernia repair than the Rives technique. However, our patients presented hernias through large orifices, with weak walls, and frequently with associated co-morbidity.
